# Emulsion-Based Gel Loaded with Ibuprofen and Its Derivatives

**DOI:** 10.3390/gels9050391

**Published:** 2023-05-08

**Authors:** Adebukola Abiola Agboola, Anna Nowak, Wiktoria Duchnik, Łukasz Kucharski, Anna Story, Grzegorz Story, Łukasz Struk, Adrian Krzysztof Antosik, Paula Ossowicz-Rupniewska

**Affiliations:** 1 Department of Chemical Organic Technology and Polymeric Materials, Faculty of Chemical Technology and Engineering, West Pomeranian University of Technology in Szczecin, Piastów Ave. 42, 71-065 Szczecin, Poland; adebukolaabiola856@gmail.com (A.A.A.); adriankrzysztofantosik@gmail.com (A.K.A.); 2 Department of Cosmetic and Pharmaceutical Chemistry, Pomeranian Medical University in Szczecin, Powstańców Wielkopolskich Ave. 72, 70-111 Szczecin, Poland; anowak@pum.edu.pl (A.N.); wiktoria.duchnik@pum.edu.pl (W.D.); lukasz.kucharski@pum.edu.pl (Ł.K.); 3 Department of Chemical and Process Engineering, Faculty of Chemical Technology and Engineering, West Pomeranian University of Technology in Szczecin, Piastów Ave. 42, 71-065 Szczecin, Poland; anna.story@zut.edu.pl (A.S.); gstory@zut.edu.pl (G.S.); 4 Department of Organic and Physical Chemistry, Faculty of Chemical Technology and Engineering, West Pomeranian University of Technology in Szczecin, Piastów Ave. 42, 71-065 Szczecin, Poland; lstruk@zut.edu.pl

**Keywords:** carbomer, increasing drug permeability, nonsteroidal anti-inflammatory drugs, ibuprofen, transdermal drug delivery, structural modification of ibuprofen

## Abstract

The aim of this study was to evaluate the effect of vehicle and chemical modifications of the structure of active compounds on the skin permeation and accumulation of ibuprofen (IBU). As a result, semi-solid formulations in the form of an emulsion-based gel loaded with ibuprofen and its derivatives, such as sodium ibuprofenate (IBUNa) and L-phenylalanine ethyl ester ibuprofenate ([PheOEt][IBU]), were developed. The properties of the obtained formulations were examined, including density, refractive index, viscosity, and particle size distribution. The parameters of release and permeability through the pig skin of the active substances contained in the obtained semi-solid formulations were determined. The results indicate that an emulsion-based gel enhanced the skin penetration of IBU and its derivatives compared to two commercial preparations in the form of a gel and a cream. The average cumulative mass of IBU after a 24 h permeation test from an emulsion-based gel formulation through human skin was 1.6–4.0 times higher than for the commercial products. Ibuprofen derivatives were evaluated as chemical penetration enhancers. After 24 h of penetration, the cumulative mass was 1086.6 ± 245.8 for IBUNa and 948.6 ± 87.5 µg IBU/cm^2^ for [PheOEt][IBU], respectively. This study demonstrates the perspective of the transdermal emulsion-based gel vehicle in conjunction with the modification of the drug as a potentially faster drug delivery system.

## 1. Introduction

Administering drugs through the skin, or transdermal drug delivery, has many advantages over other routes of administration, such as oral or injection routes [1,2,3,4,5,6]. In particular, it is a non-invasive method of drug administration that does not require punctures or incisions, which is especially important for patients who are afraid of needles [7,8]. It can also be used for patients who have difficulty taking oral medications. Transdermal drug delivery can provide continuous drug delivery over a longer period of time, which can improve therapeutic efficacy and reduce the need for frequent dosing. In addition, transdermal administration of the drug avoids the first-pass effect, which allows for higher concentrations of the drug in the blood. This route of administration allows for continuous drug delivery, which may improve patient compliance as it reduces the need for frequent dosing and may prevent missed doses. In this case, we can target the delivery of the drug to specific areas of the body, such as joints, providing local therapeutic effects. Overall, transdermal drug delivery offers a convenient and effective method of drug administration with the potential for improved therapeutic efficacy and patient compliance [1,7,9,10,11].

One of the main challenges in developing topical drug products is achieving adequate penetration of the drug through the skin. The skin is a complex barrier that provides protection against external threats but also hinders the penetration of many therapeutic agents. The outermost layer of the skin, called the *stratum corneum*, is composed of dead skin cells that are tightly packed together [8,12,13,14]. This layer acts as a barrier to the penetration of molecules larger than a few hundred Daltons, preventing the absorption of many drugs. In addition to the *stratum corneum*, other factors such as skin thickness, hydration, and the presence of hair follicles and sweat glands can also impact the penetration of drugs through the skin [15,16,17].

Various approaches have been developed to overcome these challenges and enhance drug penetration through the skin. In general, various methods of increasing the permeability of the *stratum corneum* are distinguished, including methods related to the modification of drug molecules, selection of the appropriate carrier and drug form, and incorporation of the drug into the carrier; methods related to the modification of the *stratum corneum* properties—hydration, agents that increase chemical penetration; electrically assisted methods and devices—iontophoresis, electroporation, ultrasounds; methods related to bypassing or removing the *stratum corneum*—microneedles, jet injections, and ablations [1,17,18,19].

In this publication, we will use two parallel methods related to the modification of the drug molecule and the development of the emulsion-based gel formulation.

Emulsion-based gels allow for greater stability of the resulting emulsion due to the presence of an emulsifying agent, which allows for a longer shelf life and better stability of the drug. Furthermore, this form of the drug allows for increased bioavailability, in particular for hydrophobic drugs, by increasing their solubility in the oil phase. In addition, it can prevent drug degradation in the aqueous phase. In addition, these types of formulations can increase drug penetration through the skin, allowing for a more even distribution of the drug over the skin surface and extending the time the drug is in contact with the skin. The gelling agent contained in the formulation can also control drug release over a longer period of time, providing a lasting therapeutic effect and reducing the need for frequent dosing. In addition, emulsion-based gels are easy to apply and spread evenly on the skin, providing a convenient way to administer medications [20,21,22,23,24,25].

The aim of this research will be the development of the composition of the emulsion-based gel as a ready-to-use pharmaceutical formulation and an assessment of its properties. Furthermore, these studies aim to develop a delivery system for a poorly water-soluble model drug, ibuprofen, to increase its solubility by developing a microemulsion system as a matrix and then introducing it into the gel phase. Additionally, changes in the structure of ibuprofen were also introduced in order to increase the solubility of the drug in water, and the obtained compounds were introduced into the preparations obtained for application to the skin.

## 2. Results and Discussion

The *stratum corneum*, which consists primarily of lipids, inhibits the penetration of topically applied drugs [26]. Consequently, in the penetration study, the selection of a suitable substrate may be the key to increasing penetration and achieving a rapid therapeutic effect in the subcutaneous tissues. The physicochemical properties of the compound, such as its lipophilicity, solubility, and molar mass, and the type of pharmaceutical base have a significant impact on its ability to penetrate the *stratum corneum* [27]. The ingredients of the formulation are responsible for the physicochemical properties of the pharmaceutical form and affect the penetrating ability of a given substance. The modification of the active ingredient, consisting primarily of an increase in its lipophilicity, is an additional method for enhancing penetration [28,29].

In our previous publication, we showed that amino acid alkyl ester ibuprofenates have increased skin permeability compared to ibuprofen [28,30]. The possibility of creating formulations with two commercially available, modern pharmaceutical bases—Pentravan^®^ [31] and Celugel^®^ [32]—were also tested.

As is known, the selection and use of the appropriate vehicle are crucial for the degree of absorption and therapeutic effectiveness of the drug. An ideal pharmaceutical vehicle should meet the following conditions: have the right consistency, be easy to mix with therapeutic agents, keep the active substance in constant dispersion, not react with the drug and other excipients or external factors, not cause allergies or irritations of the skin or mucous membranes, even if they are of a short-term nature, and not decompose rapidly.

### 2.1. Ibuprofenate of Ethyl L-Phenylalanine Ester

**Scheme 1:**
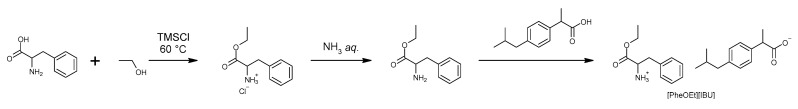
Scheme of synthesis of L-phenylalanine ethyl ester ibuprofenate used in studies.

As a result of the 3-stage reaction shown in Scheme 1, an ibuprofen derivative—L-phenylalanine ethyl ester ibuprofenate—was obtained with a total yield of 91%. The identity of the obtained compound was confirmed based on the analysis of ^1^H NMR, ^13^C NMR, and FT-IR spectra. Signal assignments, their interpretation, and all spectra are shown in Figures S1–S3. In addition, a full characterization of the obtained compound was performed.

The signal for the amino group (NH_3_^+^), confirming the ionic structure of the obtained compound, is 5.09 ppm. Furthermore, the ionic structure of L-phenylalanine ethyl ester ibuprofenate was confirmed according to the presence of two distinct absorption bands—asymmetric v(COO^−^)_as_ at 1609.07 cm^−1^ and symmetric stretching vibrations v(COO^−^)_sym_ at 1382.20 cm^−1^. The difference in frequency values between these two bands was greater than 200 cm^−1^, confirming the presence of the carboxylate anion COO^-^ and the ionic structure of the obtained ibuprofenate [30,33].

The properties of the obtained structural modification of ibuprofen ([PheOEt][IBU]) were compared with the properties of unmodified ibuprofen (IBU) and sodium salt (IBUNa).

**Figure 1:**
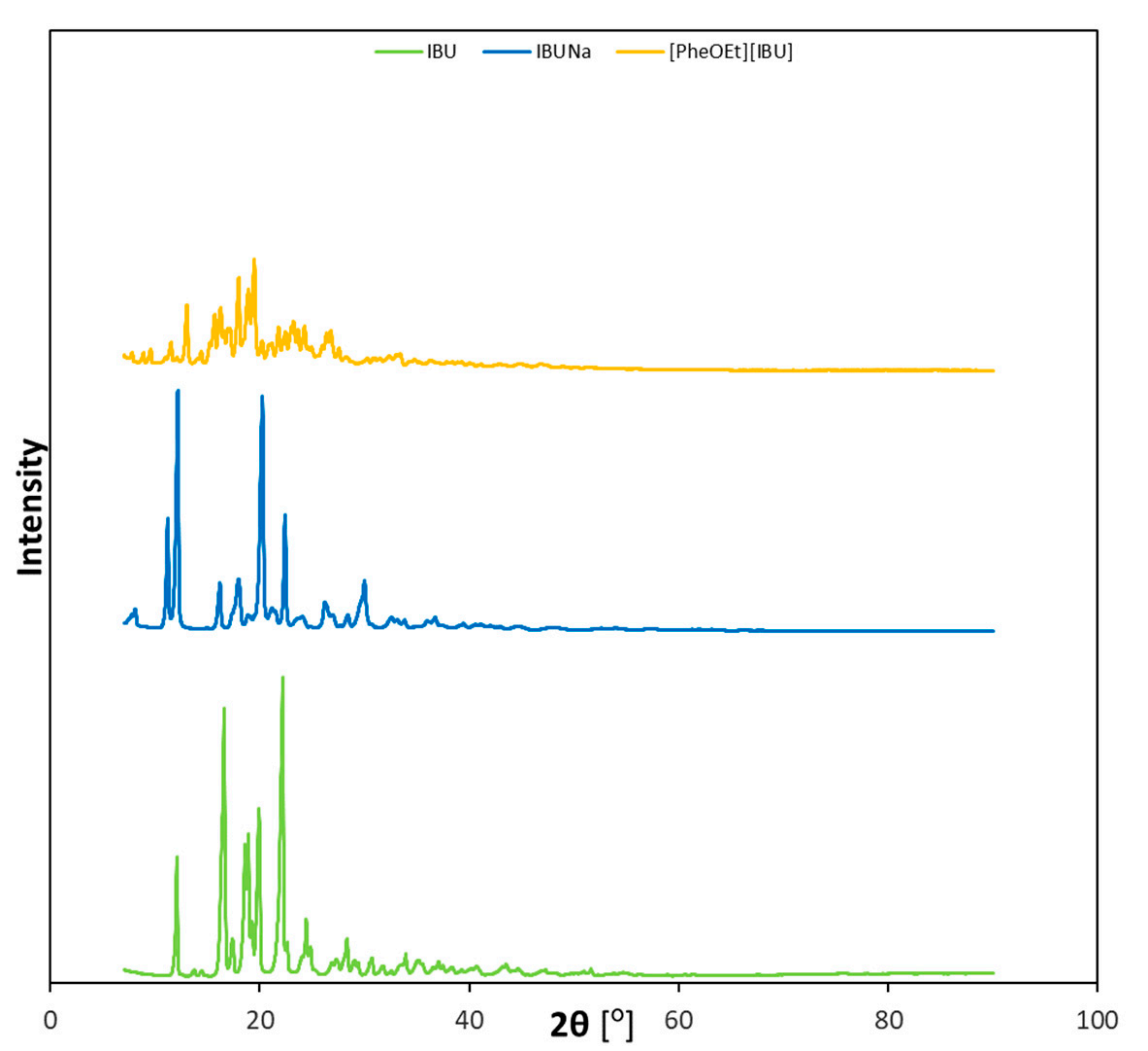
Diffractograms of IBU, IBUNa, and [PheOEt][IBU].

As can be seen in Figure 1, the diffractograms of the derivatives obtained are different from those of the starting ibuprofen. The obtained results confirmed the crystalline nature of both unmodified ibuprofen and its derivatives.

The thermal stability of drugs is an essential property necessary to control the formulation method of the finished drug. Ibuprofen, sodium ibuprofen, and L-phenylalanine ethyl ester ibuprofenate thermal stabilities were assessed and contrasted. Figure S4 displays the TG, DTG, and c-DTA curves of [PheOEt][IBU]. Both the temperature at which decomposition begins (T_onset_) and the temperature at which mass loss accelerates to its maximum (T_max_) were identified. As seen in Table 1, [PheOEt][IBU] is the least stable (Tonset = 160.8 °C), while the [IBUNa] compound is the most stable (T_onset_ = 265.4 °C). The temperatures of the decompositions of ibuprofen and ibuprofen salt are similar. However, differences in the stages of decomposition are visible. Ibuprofen decomposes in one step, [PheOEt][IBU]—in two stages, and IBUNa—in several stages.

The melting point was determined from the DSC curves. The melting point for IBU is 79.6 °C, and for [PheOEt][IBU] it is 82.3 °C. It demonstrates that the obtained [PheOEt][IBU] has a higher melting point than IBU. Since L-phenylalanine ethyl ester ibuprofenate has an ionic structure, which has already been confirmed, and a melting point below 100 °C, it can be classified as an ionic liquid. As is well known, obtaining ionic liquids has several advantages, including avoiding the phenomenon of polymorphism.

The solubility of a substance in water is used to assess the bioavailability and effectiveness of a drug. Therefore, the solubility of IBUNa (9.297 ± 0.672 g/L; 8.402 ± 0.607 g IBU/L) and [PheOEt][IBU] (0.716 ± 0.080 g/L; 0.370 ± 0.045 g IBU/L) was determined and compared to the solubility of ibuprofen (0.076 g/L). As can be seen, converting the drug to a salt form increases the solubility of the drug in water, which is a common method.

Lipophilicity is essential for predicting the hydrophobicity and partitioning of drugs in biological systems. Therefore, the test was prepared using the shake-flask method. IBU and [PheOEt][IBU] showed a positive log *p* of 2.758 ± 0.014 and 1.336 ± 0.046, respectively. On the other hand, IBUNa’s negative log *p*-value (−0.341 ± 0.176) indicates it is the most hydrophilic. Therefore, [PheOEt][IBU] is more hydrophilic than IBU but less hydrophilic than IBUNa.

### 2.2. Pre-Formulation Studies: Selection of Formulation Components

Pre-formulation studies are essential to selecting the best emulsion composition and the ingredient in which ibuprofen is most soluble. The results of ibuprofen solubility in various oils and surfactants are summarized in Table 2. It was shown that among the tested oils, ibuprofen was the most soluble in hemp oil (95.44 mg/mL), while among hydrophilic surfactants, Tween 60 (519.63 mg/mL). Furthermore, Span 80 was selected as the lipophilic surfactant, and the solubility of ibuprofen was 56.89 mg/mL. Furthermore, no interaction was observed between ibuprofen and the formulation ingredients, i.e., unplanned and unintended changes in the physicochemical properties (e.g., appearance, smell, and colour). This means that the chosen surfactant and auxiliary substances are the most appropriate for the formulation of an emulsion-based gel.

### 2.3. Preparation of Base Formulations

As a result, four emulsions with different ratios of the water phase to the oil phase were obtained (Table 3), which resulted in different amounts of surfactants being used. The ratio of hydrophilic and lipophilic surfactants was determined for the required HLB of the oil.

The stability of each of the four obtained formulations was studied at an elevated temperature of 40 °C and in a freeze-thaw stability test. There were no physical changes in color, and the appearance of F1, F2, and F3 then changed by showing separate physical phases after seven days of the testing period, leaving F4 to be the emulsion without any physical change in appearance. In the freeze-thaw test, the remaining emulsions stratified after the first cycle. In the freeze-thaw test, the emulsions F1–F3 stratified after the first cycle. The F4 emulsion was stable in all five cycles. Therefore, F4 was the most suitable formulation.

### 2.4. Preparation of Formulation with Carbomer

Since all the base formulations had inadequate consistency and were low-viscosity liquids, it was decided to refine the composition of the formulation. In order to prepare a suitable formulation, a formulation containing carbomer as a rheology-improving agent and triethanolamine as a pH regulator was developed. As a result of the work, a formulation with the desired consistency was obtained, similar to commercial pharmaceutical gel preparations for use on the skin.

### 2.5. API-Loaded Emulsion-Based Gel Formulation

According to the preparation of the emulsion, the active pharmaceutical ingredient (IBU, IBUNa, and the obtained [PheOEt][IBU]) was added, and there was no physical change in color or appearance. However, it was noticed that the addition of IBUNa significantly reduced the viscosity of the preparation. In the case of other active compounds, no differences were noticed compared to the formulation without API.

### 2.6. Determination of Physicochemical Properties of Obtained Emulsion-Based Gel Formulations

#### 2.6.1. Microscopic Examinations

**Figure 2:**
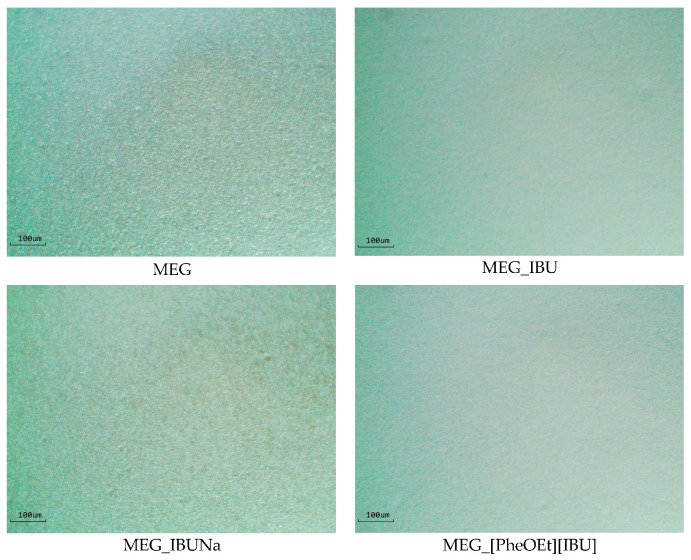
Microscopic images of (micro)emulsion-based gel loaded without and with ibuprofen and its derivatives.

The prepared formulations were observed under an optical microscope (Delta Optical, with a MC500-W3, 5 MP camera). The images presented below in Figure 2 are for the formulation without API (MEG) and with API, which contained IBU (MEG_IBU), IBUNa (MEG_IBUNa), and [PheOEt][IBU] (MEG_[PheOEt][IBU]). The result shows that the preparation was perfectly homogenous, and there are no separate phases in the formulations with different APIs.

#### 2.6.2. Density and Refractive Index

The densities of all the resulting formulations ranged from 0.9610 to 0.9855 g/cm^3^, as seen in Table 4. This result shows that the gel has a relatively lower density than water. This is due to the fact that the oil droplets in the gel are less dense than the continuous water phase, which results in a lower overall density. Compared to MEG_IBUNa, which had the highest density value, MEG_IBU displayed the lowest density. The density values are similar overall.

The refractive indices for all the resulting formulations ranged from 1.3952 to 1.4129. Compared to MEG_IBU, which has the highest refractive index value, MEG_IBUNa has the lowest refractive index. There is a negligible difference between the refractive index values of each MEG with and without API. The obtained formulations were transparent, indicating very small particle sizes. The refractive index of the formulations was just a little bit higher than that of water (1.3325).

#### 2.6.3. Viscosity

**Figure 3:**
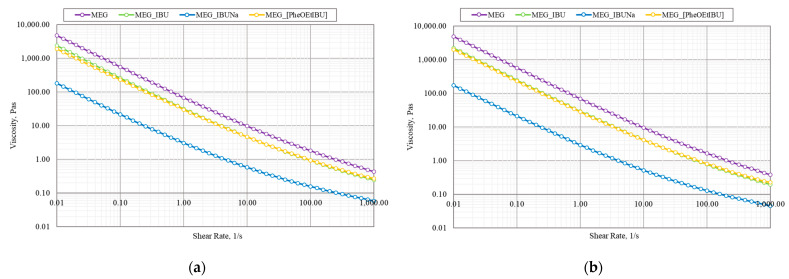
The viscosity profile of MEG_IBU, MEG_IBUNa, and MEG_[PheOEt][IBU] at temperatures (**a**) 20 °C and (**b**) 36.6 °C.

Figure 3 shows that the emulsion-based gel formulations exhibited non-Newtonian shear-thinning pseudoplastic flow. From the utility point of view, the most important are the values of the flow limit at 20 °C, which can be associated with the administration of the formulation; the viscosity at low (10–100 s^−1^) shear rates accompanying the application of the formulation on the skin; and the viscosity at shear rates in the range of 10^2^–10^3^ s^–1^ at body temperature, which characterize the rubbing of the formulation. The highest viscosity is found in MEG. However, the viscosity of the MEG decreases when API is added. The viscosities of the obtained formulations for MEG_IBU and MEG_PheOEtIBU are similar but two times lower than MEG. When IBUNa is added, the viscosity of the formulation decreases ten times more. The same relationships were found for the two temperatures used (20.0 and 36.6 °C). Was chosen as the application temperature of 20.0 °C because it is the typical storage temperature, and 36.6 °C is the typical body temperature. It can also be seen that the viscosity decreases slightly as the temperature rises.

#### 2.6.4. Particle Size Distribution

**Figure 4:**
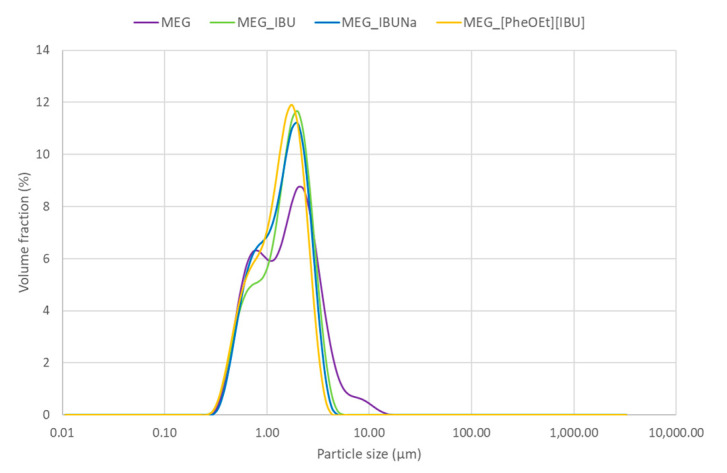
The particle size profile of MEG_IBU, MEG_IBUNa, and MEG_[PheOEt][IBU].

The particle size distribution of the emulsion-based gel was determined by a particle size analyzer. Figure 4 shows the particle size distribution of MEG with and without API expressed as a volume fraction (%). The emulsion droplets range from 0.3 μm to 19.89 μm for MEG, 0.3 μm to 6.3 μm for MEG_IBU, 0.3 μm to 5.6 μm for IBUNa, and 0.2 μm to 4.9 μm for [PheOEt][IBU]. The highest volume fraction for particle sizes of 2.0 μm was for MEG, MEG_IBU, and MEG_IBUNa, compared to MEG_[PheOEt][IBU], for which it was 1.8 μm. This range indicates that the MEG with and without API is of very small globule size. The addition of IBU, IBUNa, or [PheOEt][IBU] affects the size and distribution of the droplets or particles in the emulsion-based gel. This results in a narrower size distribution, as the droplets or particles are less likely to aggregate and form larger sizes.

#### 2.6.5. Release of the Active Substance from the Drug Form

**Figure 5:**
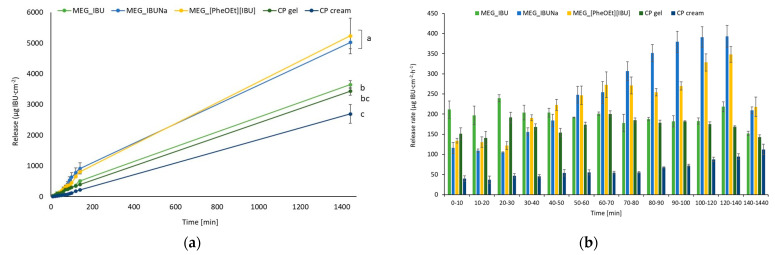
Time course of the cumulative mass of IBU and its derivatives during the 24 h release (**a**) and permeation rates of IBU and its derivatives from the emulsion-based gel through pig skin (**b**). Values are the means with a standard deviation; n = 3. The statistically significant difference was estimated using the ANOVA test.

**Figure 6:**
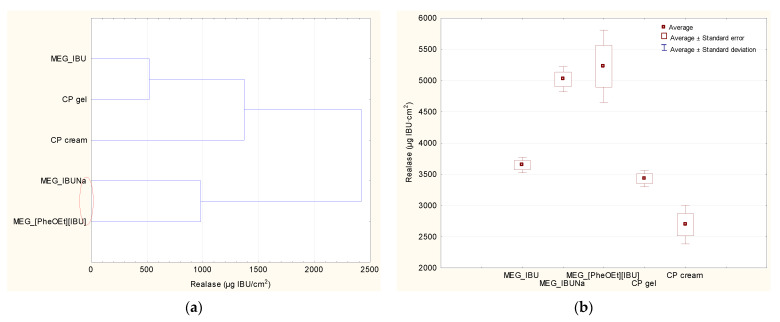
The cluster analysis graph (**a**) and the box plot (**b**) for the cumulative mass of ibuprofen and its derivatives after a 24-h study of release. The preparations with the highest release are marked with a red circle.

The release of the API from the emulsion-based gels was also determined. For comparison, the release of ibuprofen from two commercial formulations in the form of gel and cream was also carried out. Figure 5 depicts the cumulative release in μg IBU/cm^2^ as a function of time and the permeation rates of ibuprofen and its derivatives from the emulsion-based gel through pig skin. The first 180 min show the greatest release of API, which is then inhibited. As a result, the MEG formulations obtained limit the amount of active ingredient released over time. The highest statistically significant release was observed for the two ibuprofen derivatives placed in MEG formulations (Figure 6). In the release study, a statistically significant difference was observed between IBU derivatives (MEG_IBUNa and MEG_[PheOEt][IBU]), pure IBU (MEG_IBU), and two commercial preparations. No statistically significant difference was observed between MEG_IBU and CP gel. The lowest statistically significant release was observed for the commercial preparation of CP gel.

#### 2.6.6. Permeation of the Active Substance through the Skin

**Figure 7:**
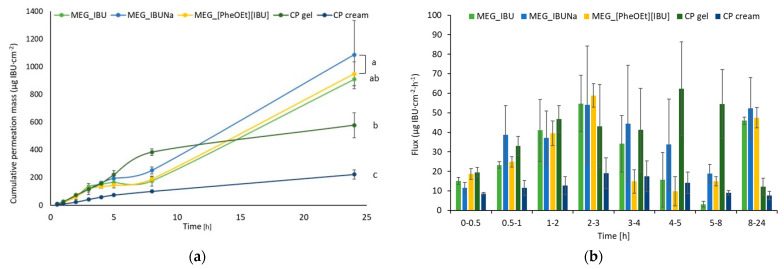
Permeation profiles (**a**) and permeation rates (**b**) of ibuprofen and its derivatives from the emulsion-based gel through pig skin. The values are the means with standard deviation; n = 3. A statistically significant difference was estimated using the ANOVA test.

**Figure 8:**
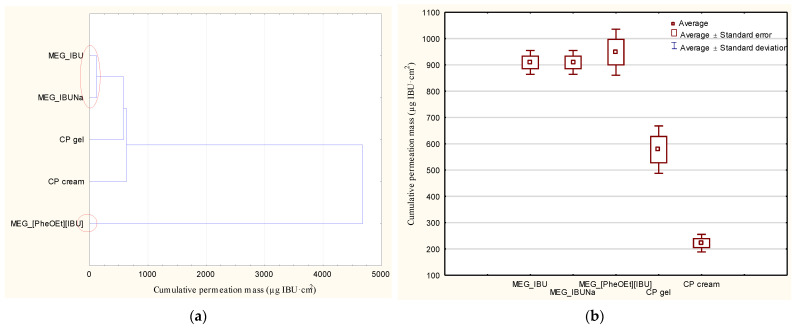
The cluster analysis graph (**a**) and the box plot (**b**) for the cumulative mass of ibuprofen and its derivatives after a 24 h study of permeation. The preparations with the highest penetration are marked with a red circle.

Figure 7 depicts individual permeation profiles for ibuprofen and its derivatives. Table 5 summarizes the cumulative mass of ibuprofen after 24 h of permeation measurement. MEG_IBUNa had a higher permeability than MEG_IBU and MEG_[PheOEt][IBU]. The total amount of substance that permeated over the course of the 24-h study was, in this case, 1086.6 ± 245.8 µg IBU/cm^2^ (Table 5). The formation of structural modifications of ibuprofen in charged compounds with lower lipophilicity has an influence on better permeability compared to more lipophilic ibuprofen. After 24 h of permeation, the cumulative mass of ibuprofen derivatives in the acceptor phase was significantly higher than that of ibuprofen and significantly higher compared to commercial preparations (Figure 8). In the pig skin penetration study, a statistically significant difference was found in the μg IBU/cm^2^ of testing for IBU derivatives (MEG_IBUNa and MEG_[PheOEt][IBU]) in comparison with commercial preparations. However, there was no significant difference between pure IBU (MEG_IBU) and CP gel. The lowest penetration, statistically significantly different, was observed for the commercial formulation of CP cream.

The permeation parameters were obtained from the typically J-shaped profiles using Equations (1) and (2) and are listed in Table 5. The lowest ibuprofen cumulated mass was determined for a commercial product in the form of cream, and it was 222.2 ± 33.3 μg IBU/cm^2^. The commercial preparation in the form of a cream used in the study was based on triglycerides of saturated fatty acids, glycerol monostearate, macrogol-30-glycerol monostearate, macrogol-100-glycerol monostearate, and propylene glycol. While for the commercial preparation in the form of a gel, the cumulative mass value was higher and amounted to 577.9 ± 90.2 μg IBU/cm^2^. In this case, the commercial preparation used is a gel based on isopropyl alcohol, 2,2-dimethyl-4-hydroxymethyl-1,3-dioxalan, poloxamer 407, and triglycerides of saturated fatty acids. In the case of the obtained emulsion-based gel formulation, a higher value was obtained for ibuprofen, and it was 909.6 ± 45.3 μg IBU/cm^2^. As can be seen, the use of sodium salt and phenylalanine ethyl ester had a beneficial effect on the amount of ibuprofen permeated. In this case, it was 1086.6 ± 245.8 and 948.6 ± 87.5 μg IBU/cm^2^, respectively. The remaining permeability parameters were comparable, and only the cream-based commercial sample showed very low values for all parameters. The highest percentage of the dosed dose was obtained using preparations containing ibuprofen salts: MEG_IBUNa and MEG_[PheOEt][IBU], which resulted in an almost fivefold increase in the permeability of the active substance. In both commercial preparations used in the comparative studies, the producers used additional promoters for the penetration of the active substance through the skin. In both cases, they were essential oils—lavender and orange oil, due to the high content of terpenes. Compared to the obtained formulations, in which no additional penetration promoters were used, drug transport through the skin was enhanced due to changes in the drug structure and/or appropriate selection of formulation ingredients.

#### 2.6.7. Stability of Obtained Formulations

The stability of each formulation obtained with different APIs (IBU, IBUNa, [PheOEt][IBU]) was studied at an elevated temperature of 40 °C in a freeze-thaw stability test. There were no physical changes in color or appearance of each formulation with a different API during the seven days of the testing period and the five freeze-thaw cycles.

## 3. Conclusions

The selection and use of the correct vehicle are crucial for the degree of absorption and therapeutic effectiveness of the drug. An ideal vehicle should meet the following conditions: have the right consistency, be easy to mix with therapeutic agents, keep the active substance in constant dispersion, not react with the drug or other excipients or external factors, not cause allergies or irritations of the skin or mucous membranes, even if they are of a short-term nature, and not decompose rapidly. It was found that the use of transdermal carriers with a modified drug molecule can be an excellent method of increasing the permeability and achieving a faster therapeutic effect, as well as a method of supplementing the action of ibuprofen with the action attributed to amino acids. As a result of the work, emulsion-based gel formulations with increased permeability of the active substance through the skin were obtained, both in comparison to commercial preparations and analogous formulations containing unmodified ibuprofen. It has been shown that the permeability of the active compound through the skin can be controlled by structural changes in the active substance and by the appropriate selection of the formulation base. It was established that to obtain drugs with increased permeability through the skin, it is most advantageous to combine various methods of increasing permeability, including drug molecule modifications and the selection of the appropriate drug form.

## 4. Materials and Methods

### 4.1. Materials

All reagents used in the study were commercially available and used without prior purification. L-Phenylalanine (99%) was provided by Alfa Aesar (Ward Hill, MA, USA). Chlorotrimethylsilane (TMSCl) (99%), ibuprofen sodium salt (98%), and acetonitrile for HPLC (99%) were provided by Sigma-Aldrich (Darmstadt, Germany). Pol-Aura (Morąg, Poland) provided ethanol (96%). The ammonium hydroxide (25%) was provided by P.P.H. Stanlab (Lublin, Polska) sp. j. Diethyl ether (99.5%) and anhydrous sodium sulfate (Na_2_SO_4_) (99%) were provided by Chempur (Piekary Śląskie, Polska). Ibuprofen (98%) was supplied by AmBeed (Arlington Heights, IL, USA), chloroform (≥99.9%) and triethanolamine (≥99.9%) were supplied by Eurochem BGD sp. z.o.o (Tarnów, Poland), and Efavit provided hemp oil. Span 80 and Tween 60 were purchased by Croda. Carbomer (Carbopol^®^ 940 NF Polymer) was purchased from Lubrizol (Wickliffe, OH, USA). The commercial products (in gel and cream form) were purchased (Dolorgiet Pharmaceuticals, Sankt Augustin, Germany).

### 4.2. L-Phenylalanine Ethyl Ester Ibuprofenate Synthesis

L-Phenylalanine ethyl ester ibuprofenate [PheOEt][IBU] used for the research was synthesized using the methodology described in our previous publication [30]. In the studies, an Ibuprofen derivative in the form of an ion pair in which the anion was ibuprofenate and the cation was the ethyl ester of L-phenylalanine. The synthesis and structure of the compound used in the research and its acronym are presented in Scheme 1.

### 4.3. Pre-Formulation Studies: Selection of Formulation Components

Based on ibuprofen solubility studies in individual components, the compounds used to produce the formulation were selected. The following oils were used in the research: hemp oil, evening primrose oil, and sunflower oil, as well as the following surfactants: Tween 40, Tween 60, Span 80, and Tween 80. For this purpose, 1 mL of the appropriate ingredient was added in portions of 100 mg ibuprofen, obtaining a supersaturated solution. The study was carried out in screw-cap vials with continuous magnetic stirring at 100 rpm at 25 °C for 48 h. A supersaturated solution was obtained and filtered through a syringe filter with MCE (Mixed Cellulose Ester) membrane (0.45 µm, ⌀ 47 mm). The content of the compound in the solution was determined by HPLC. The HPLC analyses were performed using a Shimadzu (Kyoto, Japan) Nexera-i LC-2040C 3D High Plus liquid chromatograph equipped with a DAD/FLD detector and Kinetex^®^ F5 column 100 Å (2.6 mm; 150 × 4.6 mm; Phenomenex, Torrance, CA, USA) maintained at 35 °C. The mixture of acetonitrile and water 60/40 (*v*/*v*) was used as the mobile phase under isocratic conditions, with a flow rate of 0.5 mL/min. The detection wavelength was 210 nm. The collected data were acquired and processed using LabSolutions/LC Solution System. Each measurement was performed in triplicate, and the results were averaged. The concentrations of ibuprofen and its salts were calculated based on peak area measurements using the calibration curve method.

### 4.4. Preparation of Base Formulations

A total of four emulsions were formulated, and a stability test was carried out to choose the best formulation out of the four emulsions. Table 3 shows the amount and mass of each ingredient. The amounts of surfactants were selected based on the HLB of the oil layer (HLB = 9) and the amount of oil phase used.

Hemp oil was combined with Span 80 to make the oil phase, while water and Tween 60 were combined to form the water phase; both mixtures were heated to 80 °C. The water phase was stirred with a homogenizer provided by Janke and Kunkel IKA Labortechnik at 8000 rpm. Then the oil phase was slowly added to the water phase, and both phases were vigorously mixed until the temperature of the mixture dropped to 30 °C and an emulsion was formed.

### 4.5. Preparation of Formulations with Carbomer

According to the result of the stability test, formulation 4 (F4) was determined to be the most stable and was therefore selected for further study. Due to the very low viscosity of the formulation (the resulting formulation was pourable at room temperature, like water), it was decided to change the rheology of the sample by adding an appropriate rheological regulator, carbomer 940. For this purpose, the oil layer and water layer of the emulsion were prepared analogously to the base preparation (F4), with the difference that 0.17 g of carbomer 940 was added to the aqueous layer. The amounts of ingredients used are shown in Table 6 (MEG). After both phases of the emulsion had been heated to 80 °C, the oil phase was added in portions to the water phase while stirring. Stirring was continued until the resulting emulsion cooled to below 30 °C. Then, 0.2 g of triethanolamine was added to the resulting formulation as a pH regulator.

### 4.6. API-Loaded Emulsion-Based Gel Formulation

Formulations containing pharmaceutically active ingredients (IBU, IBUNa, and [PheOEt][IBU]) were then prepared. The formulations were prepared analogously, as described in Section 4.5. API was added to the finished formulation, and then the system was mixed using a homogenizer. Table 6 shows the composition of formulations loaded with different APIs. The active substance was used at 5%, calculated as ibuprofen, comparable to commercially available preparations. Ibuprofen, sodium ibuprofenate, and L-phenylalanine ethyl ester ibuprofenate were used as active substances.

### 4.7. The Microscopic Examinations

The prepared formulations were observed under an optical microscope (Delta Optical, with a MC500-W3 5 MP camera). The camera attached to the microscope captured images at magnifications of 25×. The measurement was performed at room temperature.

### 4.8. Stability of Base Formulations

A stability test was conducted to select the most stable formulation. The first test was carried out by placing a sample of the preparation on a magnetic stirrer with a speed of rotation of 250 r/min at a temperature of 40 °C for 7 days. During the measurement, the behavior of the sample was monitored organoleptically, including color change and the separation of individual phases of the emulsion.

Another stability test was the freeze-thaw stability test. This stability test was performed by freezing the sample at about −4 °C for 24 h and then allowing the sample to thaw at room temperature for 24 h. The freezing-thawing process was repeated five times. During the measurement, the behavior of the sample was monitored organoleptically, including color change and the separation of individual phases of the emulsion.

### 4.9. Density

The density of formulations with and without API was measured using a densimeter (Densito 30px from Mettler Toledo). The test was performed at ambient temperature, repeated five times, and the value was averaged.

### 4.10. Refractive Index

Refractive index measurements were used to evaluate the four emulsions: those without API and those with API. One drop of each emulsion was placed on a slide, and refractive indices were measured using a refractometer (Refracto 30GS, Mettler Toledo). The test was repeated five times, and the value was averaged.

### 4.11. Viscosity

The rheological measurements of flow curves and apparent viscosity were made using a rotational rheometer, MCR 102 (Anton Paar), equipped with a cone-plate measuring system (CP50-1/S), with a sandblasted surface and roughness ranging from 4 to 7 μm. The sandblasted measuring spindle was used to avoid slipping the sample on its surface. The spindle diameter was 50 mm, the cone angle was 0.994°, and the taper was 103 μm. The test sample size was 0.57 mL.

During the rheological tests, the temperature of the samples was kept constant using the Peltier temperature module and was 20.0 or 36.6 °C, respectively. Measurements were carried out for a logarithmically increasing range of shear rates, γ = 0.01–1000 s^−1^, using 10 measurement points for each decade. In total, 51 measurement points were obtained for one measurement.

Measurements for each sample were made twice, and the presented results are the average of the two measurements. Due to the repeatability of the measurement results, a larger number of repetitions was not necessary.

### 4.12. Particle Size Distribution

A Mastersizer 3000 particle size analyzer was used for the measurements, enabling particle size measurement by laser diffraction (Malvern Instruments Ltd., Malvern, UK), equipped with a Hydro EV adapter for wet dispersion of samples. Measurements were carried out with the assumption of sphericity in accordance with the Mie theory. As a dispersant, demineralized water at a temperature of 20 °C with a refractive index of 1.33 was used, which was constantly stirred with a propeller stirrer at a constant speed of 1200 rpm.

Prior to the measurements, a standard cleaning of the apparatus based on three cycles was performed. Then, 600 mL of demineralized water was poured into a glass beaker placed in the Hydro EV attachment, automatic alignment of the system was performed, and the measurement background was recorded. The next step was to add the test sample with a known light scattering coefficient to the dispersant in a small amount, enabling the determination of obscure in the range of 5–10% (according to the manufacturer’s recommendations for such particle size). Then the measurement was performed by the red laser (632.8 nm) for 20 s and by the blue laser (470 nm) for the next 20 s. The number of repetitions for one sample was 160.

The results are presented as single curves as volume fraction (%) in relation to particle size (µm) and tabulated data for successive repetitions with the calculated mean, standard deviation, and relative standard deviation.

### 4.13. Release of the Active Substance from the Drug Form

The release was carried out utilizing the Franz diffusion cell (Phoenix DB-6, ABL&E-JASCO, Vienna, Austria) with diffusion regions of 1 cm^2^ in accordance with the modified method [34]. The diffusion areas were covered with a dialysis tubing cellulose membrane (D 9777-100FT, Sigma Aldrich), and the prepared formulation was mounted over the membrane.

The acceptor chamber, with a capacity of 10 mL, was filled with PBS (pH 7.4). In the donor chamber, 1 g of each formulation was dosed directly onto the cellulose membrane. The release medium was maintained at 37 °C, and the experiment was carried out for 24 h. After 10, 20, 30, 40, 50, 60, 70, 80, 100, 120 min, and 24 h of stirring, the samples were reported. For this purpose, aliquots of the acceptor fluid (0.5 mL) were removed, and freshly buffered samples with the same pH were added. The concentration of the compounds in the acceptor fluid was determined using HPLC.

The Smartline model 2600 UV detector, model 1050 pump, and model 3950 autosampler with ClarityChrom 2009 software made up the HPLC system (Knauer, Berlin, Germany). At 264 nm, the detector was in operation. A chromatographic column measuring 125 × 4 mm loaded with Hyperisil ODS (C18), particle size 5 µm, was employed. With a 1 mL/min flow rate, the mobile phase contained 0.02 M potassium dihydrogen phosphate, acetonitrile, and methanol (45/45/10 *v*/*v*/*v*).

### 4.14. Permeation of the Active Substance through the Skin

The permeation tests were performed using the same procedure as the release tests, except that a different membrane was used. Pig skin was used as the membrane in these studies since porcine skin has a similar level of permeability to human skin [35,36]. Fresh abdominal pig skin from the neighborhood slaughterhouse was repeatedly washed in PBS buffer, pH 7.4. The 0.5 mm of skin thickness was dermatomed, and the pieces were each 2 cm × 2 cm in size. Analogously, assessing the skin’s impedance, as previously described, was used to assess the skin’s integrity [37,38]. Only skin samples with impedances greater than >3 kΩ, which roughly correspond to the electrical resistance of human skin, were used [38]. On the skin, 1 g of each formulation was dosed. The experiment was conducted over 24 h. After 0.5 h, 1 h, 2 h, 3 h, 4 h, 5 h, 8 h, and 24 h from the start of the permeation experiment, 0.5 mL samples of the acceptor fluid were taken out, and the acceptor chamber was refilled with fresh PBS solution. The amounts of IBU and its salts in the acceptor fluid were determined using HPLC. Based on this concentration, the cumulative mass (µg IBU/cm^2^) was computed [39].

The kinetic profiling of in vitro infinite dose steady-state percutaneous absorption has been most often characterized by Fick’s Laws of Diffusion [40,41,42,43,44,45], as shown in Equations (1)–(3). The flux (in µg/cm^2^·h) through the pig skin into acceptor fluid was determined as the slope of the plot of cumulative mass in the acceptor fluid versus time.

The permeation parameters—fluxes of ibuprofen and its derivatives through the skin (*J_ss_*), the diffusion coefficient (*K_p_*), and the time required to reach steady-state permeation (lag time–*L_T_*)—were obtained from typically J-shaped profiles by using Equation (1):

(1) $$A=J_{ss}(t-L_{T})$$

where:

*A*—cumulative amount of active pharmaceutical ingredient (API) of IBU and its salts permeating into the receptor compartment [µg IBU·cm^−2^],

*J_ss_*—steady-state flux [µg/cm^2^·h],

*t*—time [h],

*L_T_*—lag time [h].

The steady-state flux was estimated from the slope of the linear portion of the plot of cumulative mass in the acceptor phase over time. The lag time (*L_T_*) was determined from the x-intercept of the linear part of the plot of cumulative permeation mass in the acceptor phase over time and was used to calculate the permeability coefficient (*K_p_*) by using Equation (2):

(2) $$K_{p}=\frac{J_{ss}}{C}$$

where:

*C*—concentration in the donor phase.

The diffusion coefficient (*D*) was calculated according to Equation (3):

(3) $$D=\frac{I^{2}}{6\cdot L_{T}}$$

where:

*I*—diffusional pathway length as a skin thickness [mm].

The skin partition coefficient (*K_m_*) was calculated according to Equation (4):

(4) $$K_{m}=\frac{K_{p}\cdot I}{D}$$

The enhancement factor (*EF*) was determined using Equation (5):

(5) $$EF=\frac{m_{c\_formulation}}{m_{c\_CP\;cream}}$$

where:

$m_{c\_formulation}$—cumulated mass for ibuprofen and its derivatives from obtained formulations [μg IBU/cm^2^],

$m_{{c\_}_{CP\;cream}}$—cumulated mass for ibuprofen from CP cream [μg IBU/cm^2^].

### 4.15. Statistical Analysis

This study used a one-way variance analysis (ANOVA) analysis. In the case of the cumulative mass, the significance of differences between individual formulations was evaluated with Tukey’s test (α < 0.05), where each derivative was compared to the control (MEG_IBU). In addition, a cluster analysis was carried out to determine similarities between all formulations tested, considering all time points. On this basis, the formulations presented a similar release and permeation. Statistical calculations were done using Statistica 13 PL software (StatSoft, Kraków, Poland).

## Figures and Tables

**Table 1:** Thermal stability results of the tested APIs.

Compound	T_m_ (°C)	T_onset_ (°C)	T_max_ (°C)	T_5%_ (°C)	T_50%_ (°C)
IBU	79.6	193.2	223.8	162.1	210.3
IBUNa	194.6	265.4	444.4	285.4	445.1
[PheOEt][IBU]	82.3	160.8	215.6	141.6	221.7

T_m_, melting point; T_onset,_ the onset temperature of the thermal degradation; T_max_, the temperature of the maximum mass loss rate; T_5%_, decomposition temperature, corresponds to 5% weight loss of the initial mass; T_50%_, decomposition temperature, corresponds to 50% weight loss of the initial mass.

**Table 2:** Solubility of ibuprofen in various oils and surfactants at 25 °C (mean ± S.D., n = 3).

	Components	Solubility (mg/mL)
Oils	Sunflower oil	65.10 ± 1.92
	Hemp oil	95.44 ± 0.03
	Primrose oil	76.74 ± 0.35
Surfactants	Span 80	56.89 ± 0.15
	Tween 40	466.13 ± 1.71
	Tween 60	519.63 ± 0.69
	Tween 80	411.10 ± 4.18

**Table 3:** Amount of ingredients in basic emulsions.

Formulation	Amount of Water (g)	Mass of Surfactant 1 (Tween 60) (g)	Mass of Surfactant 2 (Span 80) (g)	Mass of Oil (Hemp Oil) (g)
F1	25.32	2.69	2.38	25.02
F2	30.02	2.16	1.86	20.02
F3	35.02	1.62	1.40	15.01
F4	40.40	1.25	0.92	10.03

**Table 4:** Density and refractive index results of tested formulations without and with different APIs.

Formulation	Density (g/cm^3^)	Refractive Index
MEG	0.9826 ± 0.0008	1.3952 ± 0.0003
MEG_IBU	0.9610 ± 0.0006	1.4226 ± 0.0005
MEG_IBUNa	0.9920 ± 0.0009	1.3910 ± 0.0010
MEG_PheOEtIBU	0.9855 ± 0.0007	1.4129 ± 0.0006

**Table 5:** Skin permeation parameters for ibuprofen and its derivatives from obtained formulations; n = 3.

Formulation	Cumulated Mass, μg IBU/cm^2^	*J_ss_*, µg IBU/cm^2^·h	*K_p_*∙10^4^, cm/h	*L_T_*, h	*D*∙10^4^, cm^2^/h	*K_m_*	*Q*%_24 h_	*EF*
MEG_IBU	909.6 ± 45.3	43.439 ± 2.262	8.688 ± 0.452	0.461 ± 0.099	9.371 ± 2.294	0.048 ± 0.012	1.82 ± 0.09	4.09
MEG_IBUNa	1086.6 ± 245.8	43.741 ± 3.258	8.749 ± 0.652	0.422 ± 0.036	9.929 ± 0.808	0.045 ± 0.016	2.17 ± 0.49	4.89
MEG_[PheOEt][IBU]	948.6 ± 87.5	44.445 ± 3.501	8.889 ± 0.700	0.427 ± 0.081	10.009 ± 1.938	0.046 ± 0.012	1.90 ± 0.18	4.27
CP gel	577.9 ± 90.2	43.339 ± 7.893	8.668 ± 0.579	0.324 ± 0.044	12.834 ± 1.048	0.033 ± 0.008	1.15 ± 0.18	2.60
CP cream	222.2 ± 33.3	14.897 ± 1.236	2.979 ± 0.247	0.298 ± 0.024	13.926 ± 1.111	0.011 ± 0.002	0.44 ± 0.07	1.00

*J_ss_*—steady-state flux; *K_p_*—permeability coefficient; *L_T_*—lag time; *D*—diffusion coefficient; *K_m_*—skin partition coefficient; *Q*—the percentage of the applied dose; *EF*—enhancement factor.

**Table 6:** Amount of ingredients in an emulsion-based gel loaded with APIs.

Formulation	Amount of Water (g)	Mass of Surfactant 1 (Tween 60) (g)	Mass of Surfactant 2 (Span 80) (g)	Mass of Oil (Hemp Oil) (g)	API	Mass of API (g)
MEG	23.05	0.62	0.32	5.77	-	-
MEG_IBU	23.05	0.62	0.32	5.77	IBU	0.6280
MEG_IBUNa	23.05	0.62	0.32	5.77	IBUNa	0.6639
MEG_[PheOEt][IBU]	23.05	0.62	0.32	5.77	[PheOEt][IBU]	1.1760

## Data Availability

The data presented in this study are available on request from the corresponding author.

## Supplementary Materials

The following supporting information can be downloaded at: https://www.mdpi.com/article/10.3390/gels9050391/s1, Identity and physicochemical properties of the obtained compounds; Figure S1: **^1^**H NMR spectra for [PheOEt][IBU]; Figure S2: **^13^**C NMR spectra for [PheOEt][IBU]; Figure S3: The FT-IR spectra for [PheOEt][IBU]; Figure S4: XRD pattern of [PheOEt][IBU]; Figure S5: The DSC curve for [PheOEt][IBU].
