# Supplementary material for: Emulsion-Based Gel Loaded with Ibuprofen and Its Derivatives
Source: Gels. 2023 May 8;9(5):391. doi: 10.3390/gels9050391 (PMC10217311; doi:10.3390/gels9050391)
Supplement: Supplementary file 1 [file gels-09-00391-s001.zip › gels-2385932-supplementary.pdf]

Supplementary data

Emulsion-Based Gel Loaded with Ibuprofen and Its Derivatives

Adebukola Abiola Agboola [1], Anna Nowak [2], Wiktoria Duchnik [2], Łukasz Kucharski [2], Anna Story [3], Grzegorz Story [3], Łukasz Struk [4], Adrian Krzysztof Antosik [1] and Paula Ossowicz-Rupniewska [1,*]

[1] Department of Chemical Organic Technology and Polymeric Materials, Faculty of Chemical Technology and Engineering, West Pomeranian University of Technology in Szczecin, Piastów Ave. 42, 71-065 Szczecin, Poland; adebukolaabiola856@gmail.com (A.A.A.); adriankrzysztofantosik@gmail.com (A.K.A.)
[2] Department of Cosmetic and Pharmaceutical Chemistry, Pomeranian Medical University in Szczecin, Powstańców Wielkopolskich Ave. 72, 70-111 Szczecin, Poland; anowak@pum.edu.pl (A.N.); wiktoria.duchnik@pum.edu.pl (W.D.); lukasz.kucharski@pum.edu.pl (Ł.K.)
[3] Department of Chemical and Process Engineering, Faculty of Chemical Technology and Engineering, West Pomeranian University of Technology in Szczecin, Piastów Ave. 42, 71-065 Szczecin, Poland; anna.story@zut.edu.pl (A.S.); gstory@zut.edu.pl (G.S.)
[4] Department of Organic and Physical Chemistry, Faculty of Chemical Technology and Engineering, West Pomeranian University of Technology in Szczecin, Piastów Ave. 42, 71-065 Szczecin, Poland; lstruk@zut.edu.pl
* Correspondence: possowicz@zut.edu.pl; Tel.: +48-4494801

Identity and physicochemical properties of obtained compounds

The purity and identity of the L-phenylalanine ethyl ester ibuprofenate were confirmed by NMR, FT-IR, and UV-Vis methods.

The BRUKER DPX-400 Avance III HD spectrometer used to record the NMR spectra ran at 400.13 MHz for ^{1}H NMR spectra and 100.62 MHz for ^{13}C NMR spectra. Tetramethylsilane (TMS) served as the internal standard, and $CDCl_3$ was employed as the solvent. Coupling constants J are reported in Hz, and chemical shifts are given in ppm.

Thermo Fischer Scientific Nicolet 380 spectrometer model, equipped with an ATR diamond plate in transmission mode, was used to record the ATR-FTIR spectra. The spectra were recorded in transmission mode in the range of 4000 – 400 cm^{-1} at the resolution of 4 cm^{-1} and analyzed using the Omnic program version 7.3.

A Merck Spectroquant® Pharo 300 Spectrophotometer was used to gather UV-Vis spectra. The solutions were prepared with absolute ethanol at a concentration no greater than 0.03%. The

measurements were made with a 1 nm accuracy in a 10 mm quartz cell over the 190 to 400 nm wavelength range.

XRD, thermal stability (TG), phase transition temperatures (DSC), specific rotation, solubility in water, and lipophilicity were also determined.

The X-ray powder diffraction patterns measurements were carried out using an Aeris XRD diffractometer from Malvern Panalytical with Cu-Kα radiation (λ = 1.54056 Å).

Thermal stability was assessed using thermogravimetric analyses performed on a Netzsch thermomicrobalance TG 209 F1 Libra. The analysis was conducted under oxidative conditions, with a nitrogen flow (as protective gas) of 10 mL/min, an airflow of 25 mL/min, and a temperature range of 25 to 1000 °C. Samples of roughly 5 mg weight were heated at 10°/min. The onset decomposition temperature was calculated using the intersection of TG curve tangents. The temperatures associated with the quickest sample weight loss were calculated using the first derivative of the TG curve (DTG curve).

Phase transformation temperatures were registered using a DSC 250 from TA Instruments' Discovery DSC Series (New Castle, Delaware, USA). The aluminum pan with a crimped lid was loaded with the samples, which weighed 5 mg on average. The nitrogen atmosphere used for the measurements was 20 mL/min. 10 °C/min was the heating/cooling rate. The sample was first heated from 20 °C to (100)130 °C, then cooled to 0 °C. The temperatures reported by the DSC data are curve peaks, with melting points that become endothermic when heated and exothermic when cooled. Tin, zinc, lead, indium, and tin were utilized as standards to calibrate the temperature. Indium was used for enthalpy measurement calibration.

The measurement of specific rotation [α] was determined with an AUTOPOL IV polarimeter provided by Rudolph Research Analytical (Hackettstown, NJ, USA). The concentrations of compounds were 0.01 g/mL in ethanol as a solvent. The optical rotation was determined at the accuracy of 0.001 ° at 20.0 ± 0.1 °C and 589 nm wavelength in a TempTrol™ cell of 10 cm length.

For ibuprofen and ibuprofen salts in deionized water, solubility (saturation concentration) was established as the greatest amount of a material that can be dissolved at a given temperature in a unit of solvent volume. An excess compound was introduced to 1 mL of water in a screwed vial and rapidly mixed for 24 hours at 25.00 ± 0.05 °C. The supersaturated solution was then

filtered through a syringe filter at the appropriate temperature. The saturated solution was collected, diluted, and analyzed by HPLC to determine the concentration of the compounds.

The octanol/water partition coefficients of ibuprofen and ibuprofen salts were measured. First, 10 mg of the drug, carefully weighed, 5 mL of water saturated with n-octanol, and 5 mL of water saturated with n-octanol were added for this purpose. Next, the mixture was vigorously agitated at 25 °C for 3 hours before being centrifuged at 7500 rpm at 25 °C for 10 minutes to improve phase separation. The aqueous layer was decanted after centrifugation, and the concentration was measured by HPLC. The following formula was used to get the partition coefficient, log P:

$$\log P = \log c_{\mathrm{oct}} - \log c_{\mathrm{w}}$$

where C_w and C_{oct} are the concentrations (mg/L) of the drug dissolved in the aqueous layer (water) and octanol, respectively.

The following formula was used to compute the concentration of the drug dissolved in octanol:

$$c_{\mathrm{oct}} = c_0 - c_{\mathrm{w}}\ [\mathrm{mg/L}]$$

where c_0 is the overall concentration [mg/L] computed from the compound mass utilized in the experiment.

The HPLC analyses were performed by using Shimadzu Nexera-i LC-2040C 3D High Plus liquid chromatograph equipped with DAD/FLD detector and Kinetex® F5 column 100 Å (2.6 mm; 150×4.6 mm; Phenomenex, Torrance, CA, USA) maintained at 35 °C. The mixture of acetonitrile and water 60/40 (V/V) was used as the mobile phase under isocratic conditions, with a flow rate of 0.5 mL/min. The detection wavelength was 210 nm. The collected data were acquired and processed using LabSolutions/LC Solution System. Each measurement was performed in triplicate, and the results were averaged. The concentration of ibuprofen and its salts were calculated on peak area measurements using a calibration curve method ($y = 8\cdot10^{-4}x-0.7041$, $R^2 = 0.9945$).

[PheOEt][IBU] - L-phenylalanine ethyl ester ibuprofenate

^{1}H NMR (400 MHz, $CDCl_3$) δ 7.23 – 7.14 (m, H7, H8, H9, H10, H11, 5H), 7.11 – 7.05 (m, H16, H17, 2H), 7.04 – 7.00 (m, H18, H19, 2H), 5.09 (d, J = 3.4 Hz, H5, 3H), 4.08 (q, J = 7.1 Hz, H13, 2H), 3.71 (dd, J = 7.3, 5.5 Hz, H2, 1H), 3.59 (q, J = 7.1 Hz, H21, 1H), 3.00 (dd, J = 13.7, 5.5 Hz,

H4, 1H), 2.85 (dd, J = 13.6, 7.3 Hz, H4, 1H), 2.36 (d, J = 7.2 Hz, H26, 2H), 1.75 (dq, J = 13.6, 6.8 Hz, H27, 1H), 1.40 (d, J = 7.2 Hz, H23, 3H), 1.15 (t, J = 7.2 Hz, H14, 3H), 0.82 (d, J = 6.6 Hz, H28, H29, 6H). ^{13}C NMR (100 MHz, $CDCl_3$) δ 178.08 (C22), 173.20 (C1), 139.47 (C20), 136.78 (C15), 135.49 (C6), 128.30 (C7, C8), 128.26 (C9, C10), 127.58 (C11), 126.20 (C18, C19), 125.93 (C16, C17), 60.13 (C13), 54.02 (C2), 44.18 (C21), 44.01 (C26), 39.21 (C4), 29.15 (C27), 21.37 (C28, C29), 17.30 (C23), 13.10 (C14). FTIR: m(ATR): 3030; 2954; 2924; 2867; 1746; 1733; 1677; 1609; 1511; 1458; 1442; 1410; 1382; 1358; 1336; 1327; 1310; 1290; 1261; 1235; 1168; 1095; 1062; 1030; 1022; 1012; 962; 918; 884; 856; 799; 787; 755; 742; 697; 669; 636; 620; 595; 588; 541; 513; 498; 483; 451; 430; elemental analysis: calc. (%) for $C_{24}H_{33}NO4$ (399.523 g/mol) C (72.15), H (8.33), N (3.51), O (16.02), Found C (72.14), H (8.34), N (3.52), O (16.01); UV–Vis (EtOH): λ_{max} = 227.0 nm; T_m = 82.3 °C; $[\alpha]_D^{20}$ = +28.4 (c = 0.498 g/100 ml C_2H_5OH)

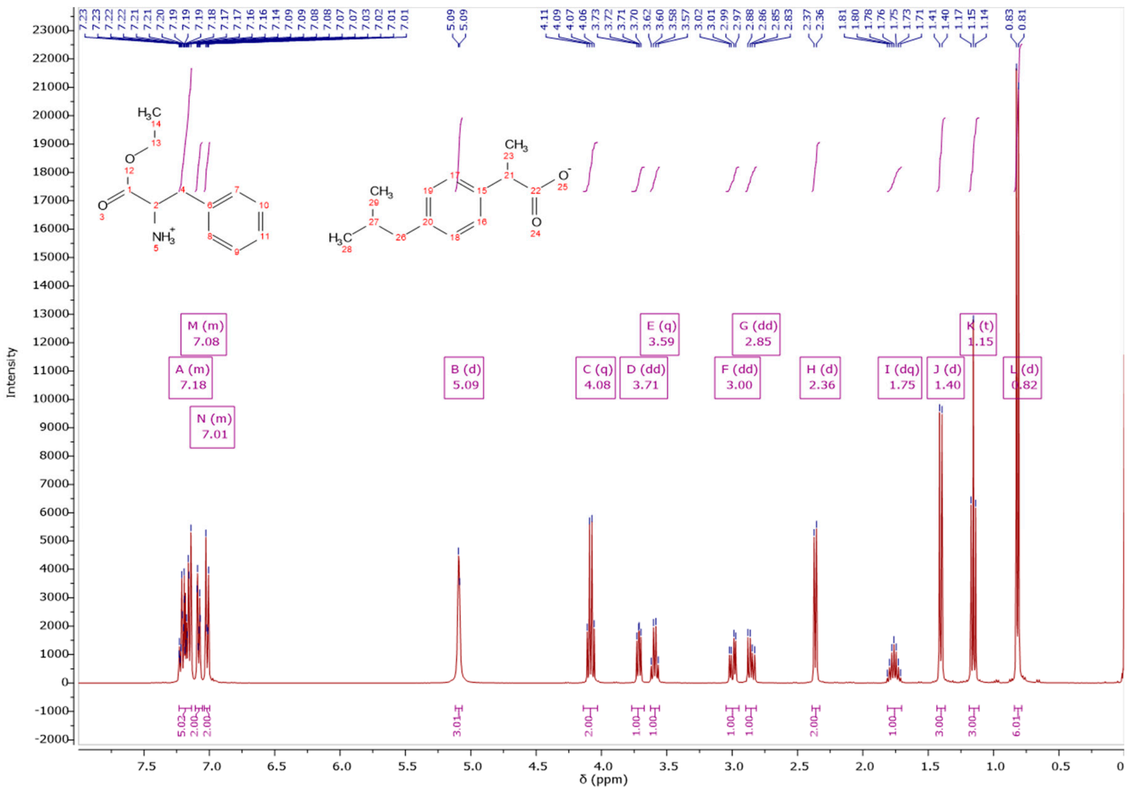

Figure S1. ^{1}H NMR spectra for [PheOEt][IBU].

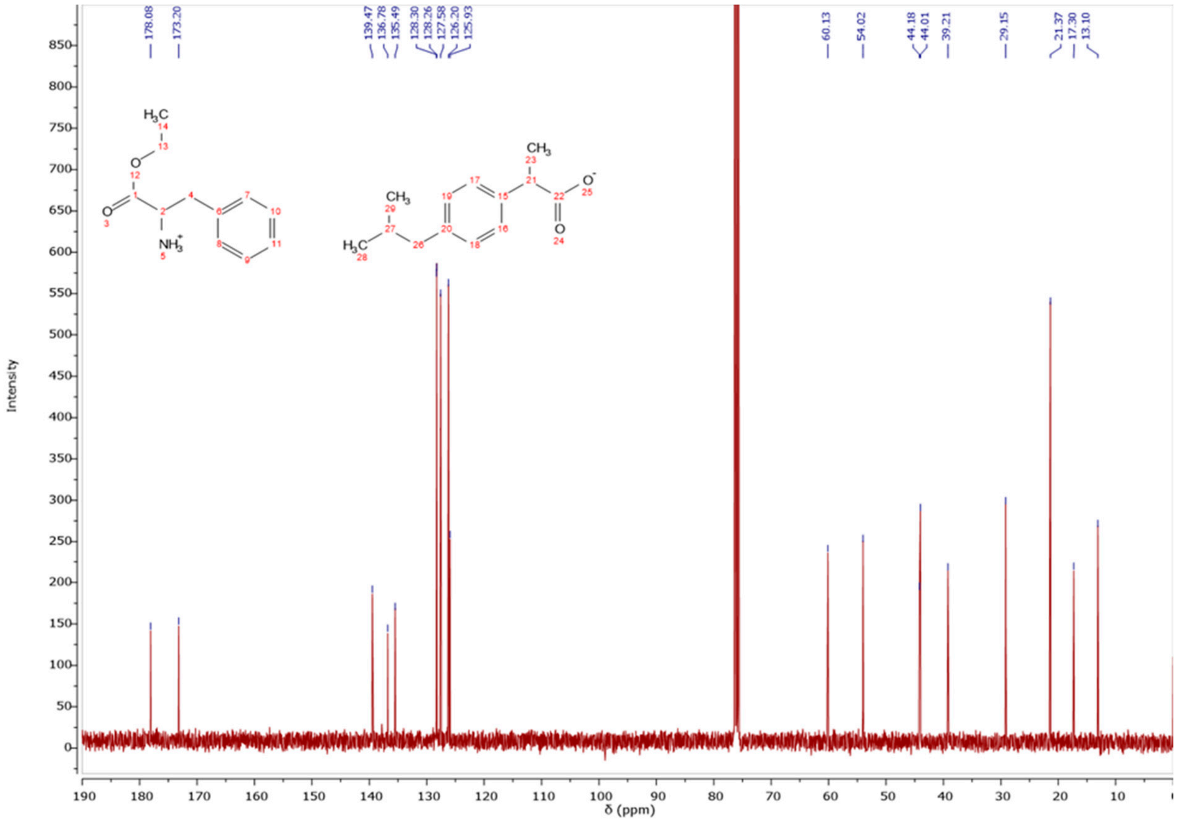

Figure S2. ^{13}C NMR spectra for [PheOEt][IBU].

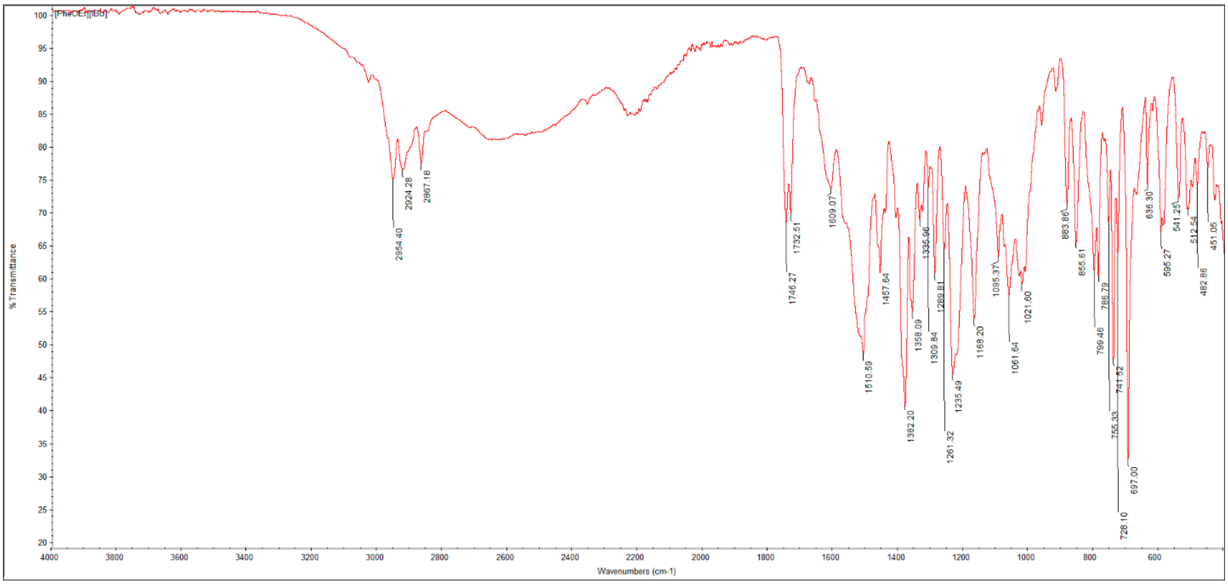

Figure S3. The FT-IR spectra for [PheOEt][IBU].

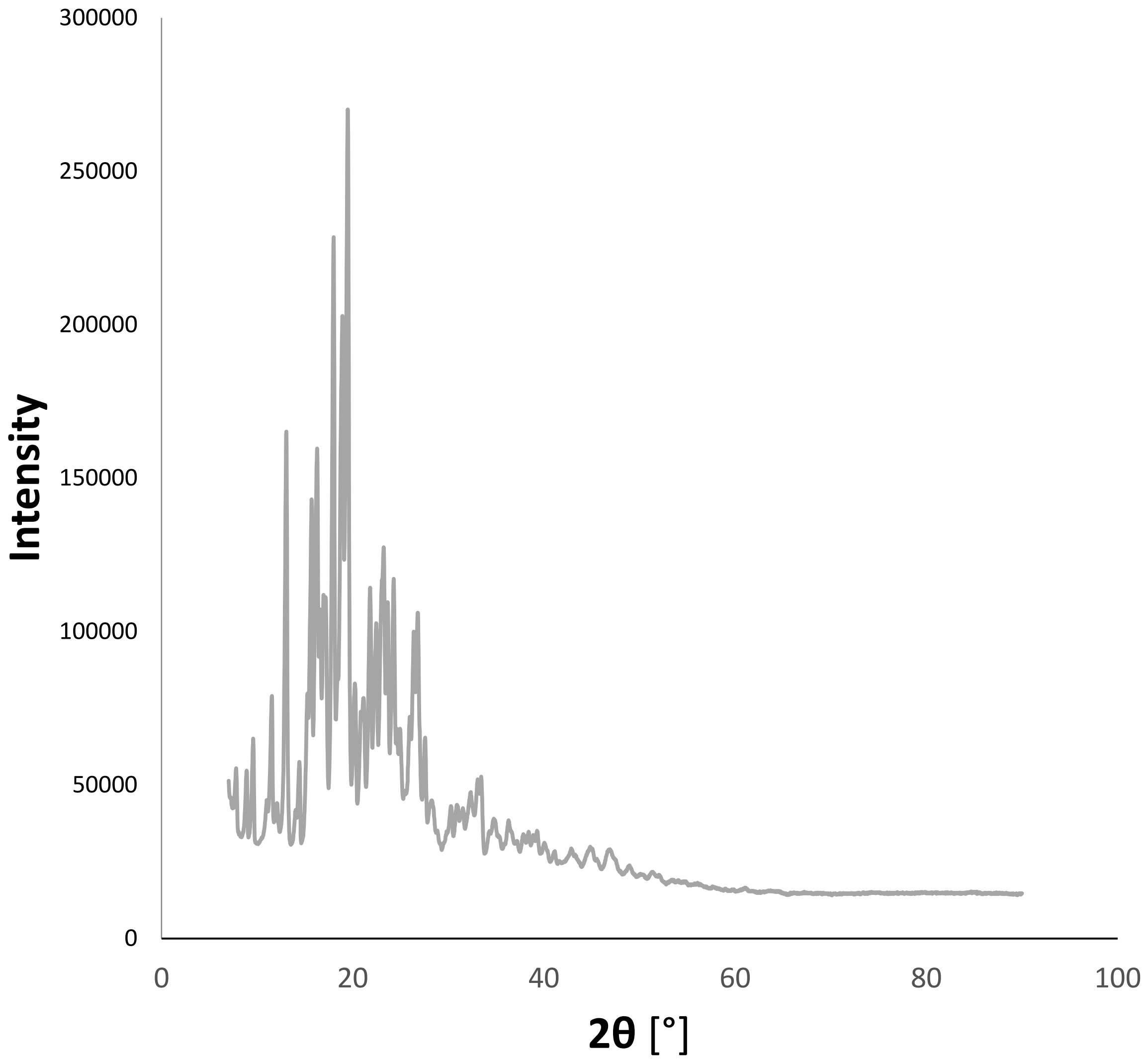

Figure S4. XRD pattern of [PheOEt][IBU].

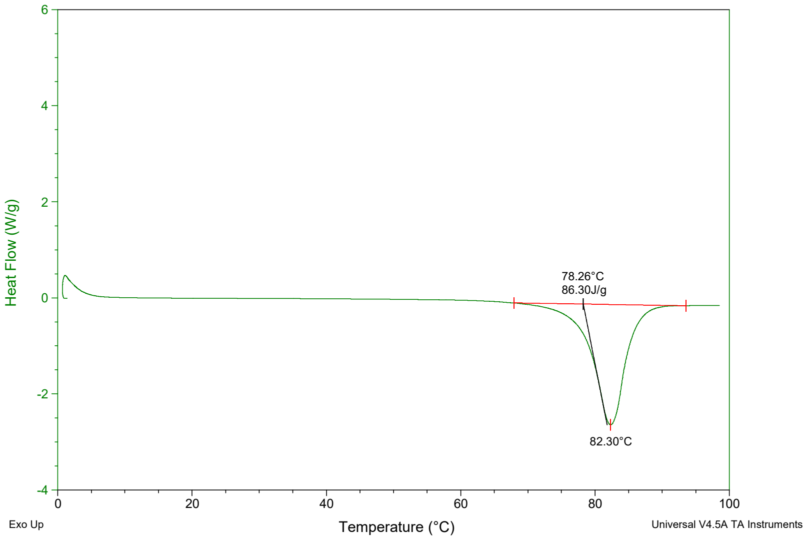

Figure S5. The DSC curve for [PheOEt][IBU].
